# A method for estimating the impact of new vaccine technologies on vaccination coverage rates

**DOI:** 10.1371/journal.pone.0263612

**Published:** 2022-02-10

**Authors:** Ben Davis, Michael Krautmann, Pascale R. Leroueil

**Affiliations:** William Davidson Institute, University of Michigan, Ann Arbor, MI, United States of America; Centers for Disease Control and Prevention, UNITED STATES

## Abstract

Vaccines are one of the most cost-effective tools for improving human health and well-being. The impact of a vaccine on population health is partly determined by its coverage rate, the proportion of eligible individuals vaccinated. Coverage rate is a function of the vaccine presentation and the population in which that presentation is deployed. This population includes not only the individuals vaccinated, but also the logistics and healthcare systems responsible for vaccine delivery. Because vaccine coverage rates remain below targets in many settings, vaccine manufacturers and purchasers have a shared interest in better understanding the relationship between vaccine presentation, population characteristics, and coverage rate. While there have been some efforts to describe this relationship, existing research and tools are limited in their ability to quantify coverage rate changes across a broad set of antigens, vaccine presentations, and geographies. In this article, we present a method for estimating the impact of improved vaccine technologies on vaccination coverage rates. It is designed for use with low- and middle-income country vaccination programs. This method uses publicly available data and simple calculations based on probability theory to generate coverage rate values. We first present the conceptual framework and mathematical approach. Using a Microsoft Excel-based implementation, we then apply the method to a vaccine technology in early-stage development: micro-array patch for a measles-rubella vaccine (MR-MAP). Example outputs indicate that a complete switch from the current subcutaneous presentation to MR-MAP in the 73 countries ever eligible for Gavi support would increase overall vaccination coverage by 3.0–4.9 percentage points depending on the final characteristics of the MR-MAP. This change equates to an additional 2.6–4.2 million children vaccinated per year. Our method can be readily extended to other antigens and vaccine technologies to provide quick, low-cost estimates of coverage impact. As vaccine manufacturers and purchasers face increasingly complex decisions, such estimates could facilitate objective comparisons between options and help these decision makers obtain the most value for money.

## Introduction

Every year, organizations spend millions of dollars on the development of new vaccine technologies such as simpler and safer administration methods and improved thermostability. These technologies may offer benefits like lower cost for vaccine storage, a reduction in the number of adverse events, and increased efficacy [1, 2]. Understanding the type and magnitude of potential benefits allows vaccine manufacturers to channel resources toward technologies with greater promise. It also allows vaccine purchasers such as donor organizations and governments to make more cost-beneficial procurement decisions. Many benefits are captured quantitatively in the context of laboratory experiments, clinical trials, and health-economic studies completed prior to product authorization. However, one benefit rarely quantified is the expected increase in vaccination coverage rates.

In this article, we propose a method for generating such estimates in a rapid and low-cost manner. This method is guided by the question, “How can publicly available data be used to estimate the impact of improved vaccine technologies on vaccination coverage rates?” While this method could be applied to any country, we focus on vaccination coverage rates in low- and middle-income countries (LMICs) for three reasons. First, while access to vaccines for childhood illnesses such as measles and polio has increased dramatically during the past 50 years, in many LMICs vaccination rates are still well below targets [3]. Second, LMICs are the types of data-limited and cost-constrained environments in which an answer that relies on data already available could be particularly helpful. Third, we expect a greater proportion of the remaining barriers to vaccination in LMICs to be technology addressable compared with high-income countries.

Although some articles in the academic literature acknowledge a relationship between new vaccine technologies and increased vaccination rates [4, 5], few provide estimates of these rates given a new technology or propose a method for generating estimates. A study in Zambia provided data on the change in measles-containing vaccine coverage given a reduction in the number of doses per vial from ten to five [6]. Other studies reported higher vaccination coverage rates when using an auto-disable syringe or a pre-filled single dose injection device [7, 8]. While informative, these studies only provide data on coverage changes for one type of technology in a single context.

In regard to methods for generating quantitative estimates of coverage rate changes, Giersing *et al*. provide a brief description of PATH’s Vaccine Technology Impact Assessment tool which estimates “the number of additional children vaccinated where a technology has the potential to increase vaccine coverage” [9]. However, additional information on this tool is not available in the literature. An abstract from Bauch *et al*. mentions a model used to explore “…the impact of new technologies under various alternative assumptions regarding how much they would increase vaccine coverage…”, but a full article describing the model is not available [10].

In this article, we first present our conceptual framework and mathematical approach. Our framework assumes that the vaccination coverage rate is a function of the vaccine presentation and the population in which that presentation is deployed, including the logistics and healthcare systems responsible for vaccine delivery. We then implement the method as a Microsoft Excel spreadsheet model and analyze a vaccine technology still in early-stage development: MR-MAP. While the example analysis includes only a single antigen and vaccine technology, our method could be readily extended to other antigens and vaccine technologies such as dual-chamber syringes and aerosolized vaccines. Finally, we discuss the benefits and limitations of our method as well as changes that could potentially improve the accuracy of outputs.

Our aim was to develop a simple method for using publicly available data to estimate the impact of new vaccine technologies on vaccination coverage rates in LMICs. We achieved this aim in terms of defining the conceptual and mathematical structure as well as implementing the method in Microsoft Excel. However, we were unable to fully validate the method due to a lack of available data. Despite this limitation, our method offers a systematic and transparent alternative to other approaches currently used to answer a similar research question. We believe it can help guide vaccine manufacturers and purchasers, both of whom face many potential options, toward more cost-beneficial vaccine technologies.

## Description of the conceptual model

### Framework

We estimate vaccination rates as determined by barriers to vaccination. For a given country or sub-national region, individuals can be modeled as facing a probability of overcoming one or more barriers. To be vaccinated, an individual must overcome all barriers. We assume that an individual can face both supply-side barriers, such as limited access to cold storage or trained medical personnel, and demand-side barriers, such as concern about the adjuvants used in a vaccine presentation. See Phillips et al. [11] for a systematic review of the determinants of effective vaccine coverage in LMICs.

Vaccine technologies that address specific barriers can increase the probability that an individual will overcome all barriers and thus increase vaccination rates. To isolate the effects of these technologies, we make a distinction between barriers that are directly addressable by vaccine technologies (“technology-addressable”) and barriers that are not (“non-technology-addressable”).

**Fig 1:**
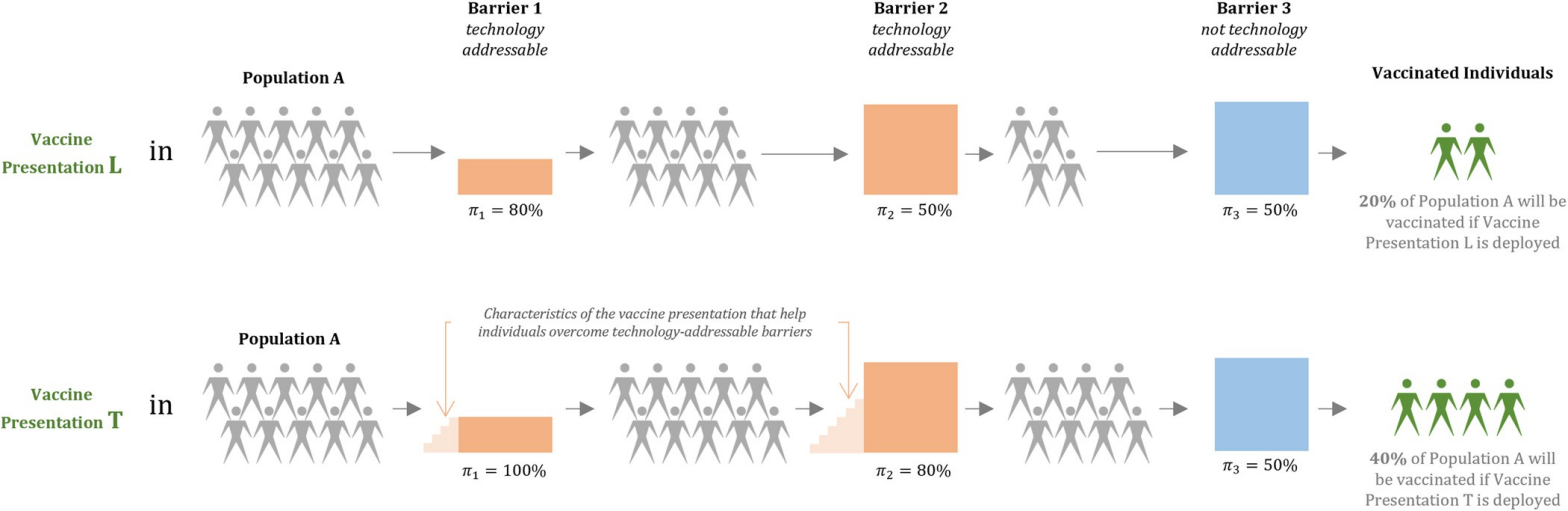
Simple example of the conceptual framework used to estimate vaccination coverage.

Fig 1 provides a simplified depiction of our conceptual framework. In this example, we assume that there are a total of three barriers to vaccination: two technology-addressable barriers (dark orange) and one non-technology-addressable barrier (dark blue). Only individuals who overcome all three barriers to vaccination will be vaccinated. The magnitude of each barrier is defined by the characteristics of the population and the systems in which that population operates. For a given point in time, the magnitude of each barrier, represented by the height of the dark orange and blue bars, is fixed. Changing the magnitude of these barriers would require a long-term intervention (e.g., expanding access to electricity or updating cold chain equipment across the health system). We treat non-technology-addressable barriers as exogenous to our model.

The probability that an individual will overcome each barrier (*π*_*n*_) is a function of the magnitude of the barrier and, if applicable, any short- or medium-term interventions such as new vaccine technologies that help an individual climb over the barrier. These interventions provide “steps” that increase the probability an individual will overcome a given barrier. “Steps” are drawn in light orange next to the technology-addressable barriers.

The top half of Fig 1 describes the deployment of Vaccine Presentation L in Population A. This presentation is the “least desirable” and has no characteristics that help individuals overcome technology-addressable barriers. The probability that an individual in Population A will overcome all barriers and be vaccinated when using Presentation L is 20%, the product of the probabilities that an individual will overcome each barrier (80% * 50% * 50% = 20%). Due to a lack of data on the degree of overlap between barriers in different contexts, we assume independence for probability calculations.

In the bottom half of Fig 1, a “test” presentation with improved technology (Presentation T) is deployed in the same population instead of Presentation L. Presentation T has characteristics that help individuals overcome technology-addressable barriers. In the example shown, these characteristics increase the probability that an individual will overcome Barrier 1 from 80% to 100% and Barrier 2 from 50% to 80%. The overall probability of vaccination increases to 40% (100% * 80% * 50% = 40%).

**Fig 2:**
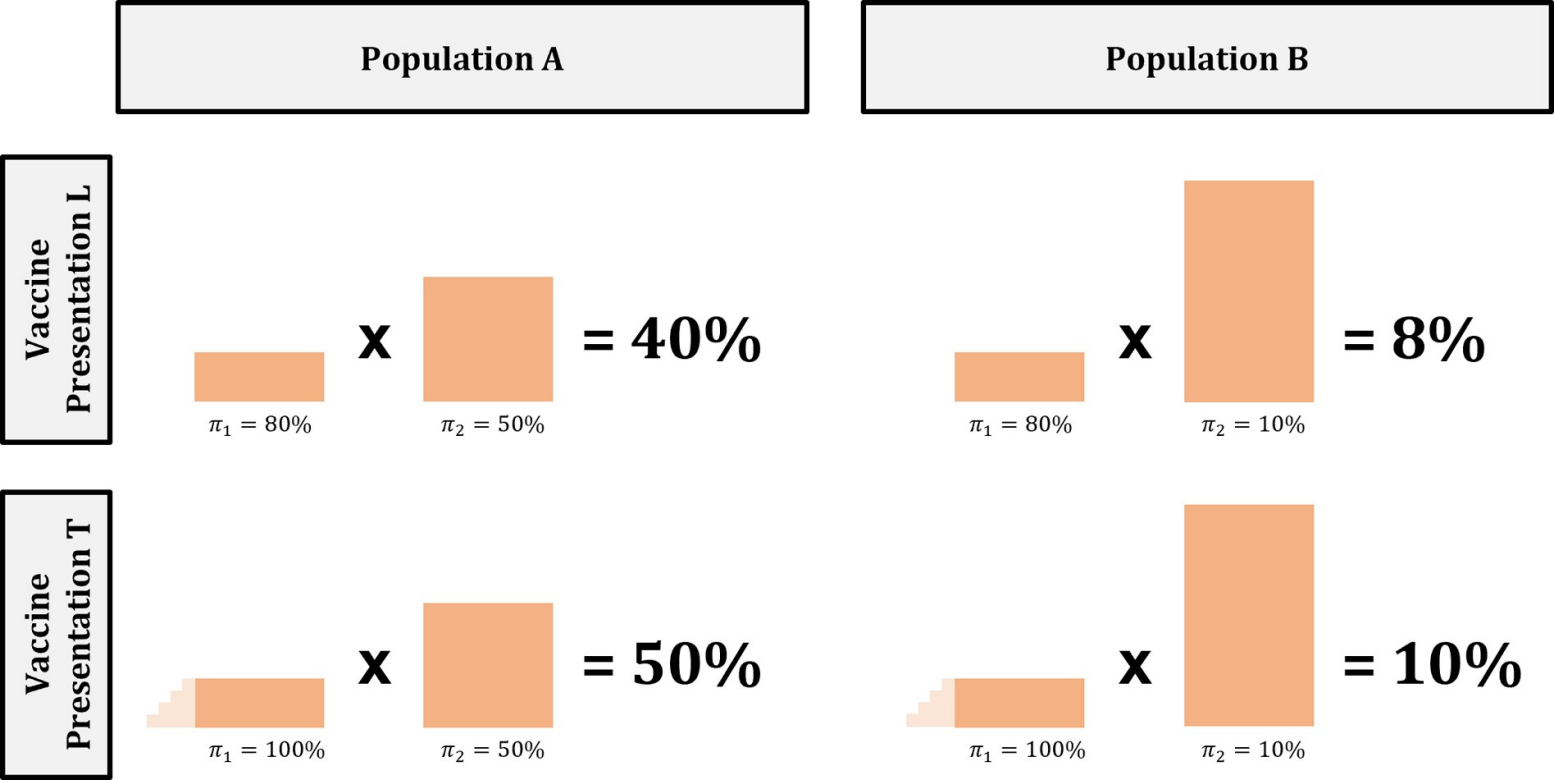
Example illustrating the importance of aligning vaccine technology with barriers faced by a population. In this example the new technology in Vaccine Presentation T affects only Barrier 1 and therefore has a lower impact in Population B, for which Barrier 2 is much more prominent.

One implication of this approach is that the change in coverage rate attributable to a new vaccine technology depends on the relative size of barriers in the population, i.e., the proportion of the vaccine-eligible population affected by each barrier. A simple example using two barriers and two populations is shown in Fig 2.

The top half of the figure illustrates the probability of overcoming both barriers when using Vaccine Presentation L. In Population A, the probability that an individual overcomes Barrier 1 is 80%, and the probability that an individual overcomes Barrier 2 is 50%. In Population B, the probabilities are 80% for Barrier 1 and 10% for Barrier 2.

The bottom half of the figure illustrates the effect of deploying a “test” vaccine with improved technology (Presentation T) in these two populations. Note that unlike the “test” presentation described in Fig 1, in this example Presentation T increases the probability of overcoming Barrier 1 to 100% but *has no effect* on Barrier 2. While the relative change in the probability of overcoming both barriers is a 25% increase in Populations A and B, the absolute change is greater in Population A (10 percentage point increase) than in Population B (two percentage point increase). This simple exercise highlights the importance of aligning the characteristics of a vaccine presentation with the barriers faced by a specific population. Misalignment could lead to a coverage rate effect that is smaller than expected.

### Mathematical approach

In this section, we present the mathematical approach developed using our framework. Eq (1) provides the general form for estimating the coverage rate of a new vaccine presentation in a given population.

$$C_{t}=M-kx_{t}$$ (1)

where:

*C*_*t*_ = estimated coverage rate of vaccine presentation *t* in a given population;

*M* = maximum coverage threshold that can be achieved only through changes to the vaccine presentation (i.e., vaccine technologies);

*k* = total percentage points of the coverage rate that can be affected by changes to the vaccine presentation;

*x*_*t*_ = probability that individuals do not overcome at least one technology-addressable barrier to vaccination when using vaccine presentation *t*.

Both *M* and *k* are constant for a given population, and *M* ≥ *k*. The value of *x*_*t*_ is between 0 and 1. This equation reflects our intuition for isolating changes in coverage due to vaccine technologies from changes due to other factors. For a given population, there is a maximum coverage threshold that can be achieved only through changes to vaccine technologies. Coverage gains beyond this threshold would require changes to the broader health system. Below we describe the calculations for each term in Eq (1).

#### Selecting *M*

*M* is the maximum coverage rate achievable only through changes in vaccine technology. It is the coverage rate that could be obtained if the “perfect” vaccine presentation were deployed in a given population. This value is an input. The user must choose *M* based on their understanding of the available data and context. We believe that a reasonable value would be the highest coverage rate of any vaccine already deployed in the population of interest. Limiting *M* to vaccines already deployed in the population of interest would reflect the current state of the logistics and healthcare systems responsible for vaccine delivery.

#### Calculating *k*

*k* is the total percentage points of the vaccination coverage rate that can be affected by changes to the vaccine presentation. It is the difference in the expected coverage rates of the “perfect” vaccine presentation (*M*) and the “least desirable” vaccine presentation (*C*_*l*_). The relationship is shown in Eq (2). The value of *k* is large for populations that face significant barriers to vaccination that can be addressed by vaccine technologies.


$$k=M-C_{l}$$ (2)

Although the concrete meaning behind *k* is relatively straightforward, the calculations behind *k* (or more precisely, *C*_*l*_ since we have defined *M* as an input above) are not. We begin by defining two vectors of dimension *n*, where *n* is the number of technology-addressable barriers (excluding the barrier specific to the number of doses required for full vaccination, “Dose Requirements”). Vector **p**, Eq (3), contains one Population Score for each technology-addressable barrier. Each Population Score describes the prevalence of a barrier in the target population. Vector **v,** Eq (4), contains one Vaccine Technology Score for each technology-addressable barrier. Each Vaccine Technology Score captures the extent to which the new vaccine technology assists the target population in overcoming a barrier to vaccination. Both Population and Vaccine Technology scores range from 0 to 100%.


$$p=(p_{1},p_{2},\ldots ,p_{n-1},p_{n})$$ (3)

$$v=(v_{1},v_{2},\ldots ,v_{n-1},v_{n})$$ (4)

Returning to our conceptual framework, the probability that an individual in a given population overcomes all technology-addressable barriers, excluding the barrier for “Dose Requirements”, is proportional to some form of the product of vectors **p** and **v.** As mentioned above, we assume that technology-addressable barriers are independent of each other. For the purpose of this model, the Population Scores within vector **p** are proportional to the probability of an individual within a given population *not overcoming* each technology-addressable barrier. Conversely, the Vaccine Technology Scores within vector **v** are proportional to that individual’s ability to *overcome* each technology-addressable barrier given the characteristics of the vaccine. Note that we chose to define the scores in this way to facilitate calculations. However, because of these definitions, the probability that an individual overcomes all technology-addressable barrier*s*, excluding the barrier for “Dose Requirements”, (*U*^*0*^) is not simply proportional to the product of vectors **p** and **v.** Instead, it is the relationship shown in Eq (5).


$$U_{vaccine}^{0}\;\alpha \prod_{i=1}^{n}\left[1-p_{i}(1-v_{i})\right]$$ (5)

We consider that “Dose Requirements” is a technology-addressable barrier. Drop-out and delayed vaccination are known risks with multi-dose vaccines, and vaccine technologies could affect the total number of doses needed for immunization. To incorporate this barrier, we assume that individuals have a constant and independent probability of receiving each successive dose of a multi-dose vaccine. If the probability of receiving one dose is *π*, the probability of receiving *D* doses is *π*^*D*^. This leads us to a more generalized form, *U*, which incorporates the “Dose Requirements” barrier. Eq (6) allows us to calculate the *uncalibrated coverage* for a given vaccine presentation (defined by **v** and *D)* in a specific population (defined by **p**).


$$U_{vaccine}={\left(\prod_{i=1}^{n}\left[1-p_{i}(1-v_{i})\right]\right)}^{D}$$ (6)

If *all* barriers to vaccination were technology-addressable barriers, and our assumptions such as the independence of barriers held, Eq (6) would be sufficient for defining coverage. If an observed coverage rate was available, we would expect alignment between *U*_*vaccine*_ and the observed rate. However, we know there are barriers that cannot be fully addressed by vaccine technologies. For example, there may be an insufficient number of vehicles to transport vaccines from central warehouses to clinics, or poor inventory management leading to frequent vaccine stock outs. These barriers limit the change in vaccination coverage possible through only changes to the vaccine presentation. Moreover, we expect that certain assumptions may not hold in the real-world, such as the independence of barriers. To address these issues, we complete a calibration step. We assume a linear relationship between calibrated coverage (*C*_*vaccine*_) and uncalibrated coverage (*U*_*vaccine*_). This relationship is shown in Eq (7), where *S* is the scaling factor (or slope) of the line between *C*_*vaccine*_ and *U*_*vaccine*_ and *β* is its y-intercept.


$$C_{vaccine}=S*U_{vaccine}+\beta$$ (7)

As described above, uncalibrated coverage is an estimate of the vaccination coverage rate accounting only for technology-addressable barriers and including assumptions such as independence of barriers. Calibrated coverage accounts for both technology- and non-technology-addressable barriers. While calibration does not relax the assumption about independence of barriers, it uses the observed coverage rate of a currently deployed vaccine to limit the magnitude of over- or under-estimation due to this assumption.

To calculate *S*, we define two points on a graph of calibrated coverage versus uncalibrated coverage. The first point we use to calculate *S* is our hypothetical ideal vaccine presentation associated with coverage *M*. The calibrated coverage value for *M* is an input, while the uncalibrated coverage value for *M* is, by definition, 100%. The second point we use is the coverage associated with a “calibration” vaccine, *C*_*c*_. This vaccine, selected by the user, must already be deployed in the target population. Since we have assumed a linear relationship, one could, in principle, use any two points in this space to calculate *S*. In practice, choosing at least one point that is relatively close to the uncalibrated test vaccine (i.e., a “calibration” vaccine with characteristics like those of the vaccine to test) will help minimize the reliance on linearity across large ranges of *C*_*vaccine*_ and *U*_*vaccine*_. Note that *C*_*c*_ is a reported coverage value and therefore does not need to be calibrated. *S* can be calculated as shown in Eq (8).

$$S=\frac{C_{2}-C_{1}}{U_{2}-U_{1}}=\frac{M-C_{c}}{{1-U}_{c}}$$ (8)

where:

*M* = maximum coverage threshold that can be achieved only through changes to the vaccine presentation *(defined input);*

*C*_*c*_ = coverage for the “calibration” vaccine *(reported value for a specific vaccine presentation in the target population);*

*U*_*c*_ = uncalibrated coverage for the “calibration” vaccine *(calculated using Eq* (6) *and the* ***p***, ***v*** *and* D *values associated with the “calibration” vaccine)*.

Having calculated the slope associated with the line between the two points, we can calculate the y-intercept, *β*, as shown in Eq (9). *β* represents the proportion of the population that would be vaccinated if the “least desirable” vaccine presentation were deployed.


$$\beta =C_{C}-S*U_{C}$$ (9)

We can convert any uncalibrated vaccine coverage rate to a calibrated vaccine coverage rate by multiplying it by the scaling factor *S* and adding the intercept *β* calculated above, as shown in Eq (7). While Eq (7) could be used to directly calculate *C*_*t*_, our value of interest, it does not allow us to easily evaluate the relationship between *C*_*t*_ and *M*, *k*, and *x*_*t*_.

Using Eq (7) or Eq (9), we can calculate *C*_*l*,_ the calibrated vaccine coverage associated with our hypothetical “least desirable” vaccine presentation. By definition, **v** in the *U* term for the “least desirable” vaccine is a vector of zeroes with length *n* because this presentation fails to help individuals overcome any barrier. *D* for the “least desirable” vaccine is the highest number of doses required by any vaccine available to achieve full protection; in the following example application, we use a value of four.

We can now write *k*, the total percentage points of the vaccination coverage rate that can be affected by changes to the vaccine presentation, in the expanded form shown in Eq (10).


$$k=M-C_{l}=M-((\frac{M-C_{c}}{{1-U}_{c}})*U_{l}+\beta )$$ (10)

#### Calculating *x*_*t*_

The variable *x*_*t*_ is defined as the probability that individuals do not overcome at least one technology-addressable barrier to vaccination when using vaccine presentation *t*. As a first approximation one might assume that this value is 1 –*U*_*t*_. However, we must account for the fact that some of the people vaccinated with the test vaccine presentation would have been vaccinated regardless of the vaccine presentation available. Recall that this probability is represented by *U*_*l*_. This leads us to Eq (11).


$$x_{t}=1-\frac{U_{t}-U_{l}}{1-U_{l}}$$ (11)

Note that there is no need to calibrate the terms in *x*_*t*_ since it will be later multiplied by *k*, which has *S* and *β* embedded.

#### Calculating *C*_*t*_

We now have all the components necessary to calculate *C*_*t*_, the estimated coverage rate with test vaccine presentation *t* for a given population, using Eq (1). The expanded form of Eq (1) is shown in Eq (12).


$$C_{t}=M-kx_{t}=M-(M-((\frac{M-C_{c}}{{1-U}_{c}})*U_{l}+\beta ))*(1-\frac{U_{t}-U_{l}}{1-U_{l}})$$ (12)

Inherent in this calculation is the assumption that enough doses of the test vaccine presentation are purchased for all vaccine-eligible individuals in the population, or that the purchased doses are perfectly targeted to individuals who will overcome their remaining barriers to vaccination through the technology in the test presentation.

### Implementation

We implemented the method described above as a spreadsheet model in Microsoft Excel (S1 File). The model includes four main worksheets. The Analysis Setup worksheet defines the target population (e.g., countries, subnational regions), technology-addressable barriers, and vaccine presentations to use for the “calibration” and “test” vaccines. The Scenario Inputs worksheet provides a table of input values for calculations that is generated based on selections made in the Analysis Setup worksheet. The Calculation worksheet uses values from the Scenario Inputs worksheet to complete the calculation steps described in the previous section. Finally, the Scenario Outputs worksheet provides a table used to calculate cross-country or cross-region estimates of vaccination coverage rates. We calculate *C*_*t*_ once for each country or subnational region selected in the Analysis Setup worksheet. Cross-country or cross-region values are an average weighted by the size of the vaccine-eligible population in each country or region.

There are several notable features of this implementation. We used Visual Basic for Applications to automate some steps, which facilitates multi-country or multi-region analysis. The user can perform sensitivity analyses concurrently with the primary analysis by selecting up to three input parameters to vary.

We also incorporated the ability to analyze two different vaccination channels simultaneously, e.g., routine immunization and supplementary immunization activities (SIAs). The user can indicate that certain technology-addressable barriers are only applicable in one of the two channels. Each calculation step is performed separately for each channel and then outputs are provided as a weighted average.

Finally, if the user performs analysis at the subnational level, the model calculates equity of coverage between regions, *E*. For this calculation, we create a vector **c** of length *r*, where *r* is the number of subnational regions of a given country included in the analysis. Each element in vector **c** is the estimated coverage rate with the “test” vaccine, *C*_*t*_, calculated for a subnational region.


$$c=(c_{1},c_{2},\ldots ,c_{r-1},c_{r})$$ (13)

$$E=1-\frac{\sum_{i=1}^{r}\left(max(c)-c_{i}\right)}{r-1}$$ (14)

The example application presented in the next section is a national-level analysis that includes only routine immunization and no sensitivity analyses.

### Validation

Through contacts at the Bill & Melinda Gates Foundation, the World Health Organization (WHO), Gavi, the Vaccine Alliance, and other global health organizations, we attempted to identify public or private data sets sufficient for a full validation of the method and implementation described above. To enable full validation, data sets needed to provide: vaccination coverage rates in a population before and after introduction of a new vaccine technology, proxy data to define Population Scores for the same population reflected in coverage rates, sufficient information to define Vaccine Technology Scores for the “calibration” and “test” vaccines, and the proportion of the population vaccinated through SIAs with each vaccine.

No single data set or combination of data sets met these criteria. We explored several technologies such as a HPV vaccine that requires three doses instead of two and a Hepatitis B vaccine that uses a compact, pre‐filled, auto-disable injection device. Issues with existing data sets included a lack of coverage data from the same population both before and after introduction of the new technology as well as ambiguous reporting (e.g., coverage rates for 3-dose and 2-dose regimens aggregated into a single statistic).

Despite a lack of data for full validation, we did receive feedback on the conceptual framework and basic calculation structure from the WHO’s Immunization Practices Advisory Committee (IPAC) [12], as well as a subgroup of Gavi’s Vaccine Innovation Prioritisation Strategy steering committee [13]. We used this feedback to refine our approach and better align the method with related initiatives at these organizations.

## Example application of method

For this example analysis, we applied the model to MR-MAP for the 73 countries ever eligible for Gavi support (“Gavi73”). We selected MAP because this technology has received considerable attention from global health donors and investors. MAPs are one of the three vaccine technologies prioritized by Gavi’s Vaccine Innovation Prioritisation Strategy [13]. A MAP could offer substantial improvements over current presentations such as the ability to forego a needle and syringe, increased thermostability, and administration by a health worker with less training than a physician or nurse.

### Setup and execution

We began by defining technology-addressable barriers to vaccination. For this list, we drew on work completed by the WHO and other partners under the Total Systems Effectiveness project [14]. We then compiled an input data set based on the technology-addressable barriers to vaccination as well as the antigens and vaccine presentations to test. For the Population Scores of each technology-addressable barrier, we created an “ideal” definition and then identified one or more proxy indicators from publicly available data sets. Proxy indicators are organized in a hierarchy. If data for a given country or subnational region are available from the Priority 1 source, these data are used for calculations. If not, the model looks for data from the Priority 2 source, then the Priority 3 source, etc. Table 1 summarizes the Population Score inputs.

**Table 1:** Definitions and sources used to assign Population Scores for each of the technology-addressable barriers developed by the WHO Total Systems Effectiveness working group.

Technology-Addressable Barrier	Ideal Definition	Definitions from Proxy Indicators
**1. Vaccine Schedule**	Probability that a member of the vaccine-eligible population does not receive vaccination due to (caregiver) inability to comply with vaccine schedule	**Priority 1:** Women who had 1–3 antenatal care visits as a % of women age 15–49 who had a live birth in the five years preceding the survey [15] **Priority 2:** Population-weighted average by World Bank Sub-region of: Women who had 1–3 antenatal care visits as a % of women age 15–49 who had a live birth in the five years preceding the survey [15, 16]
**2. Temperature Storage Requirements**	Probability that a member of the vaccine-eligible population does not have access to vaccines that have been properly stored in a functional cold chain environment since manufacture	**Priority 1**: % of total population without access to a health facility with working cold storage equipment [17, 18] **Priority 2**: 1 - [% of sampled facilities that have one or more functioning refrigerators, public facilities] [19] **Priority 3**: 1 - [% of sampled facilities that report having the electric power grid, a fuel operated generator, a battery operated generator or a solar powered system as their main source of electricity, public facilities] [19] **Priority 4**: 1 - [Access to electricity, (% of population)] [20]
**3. Administration Requirements**	Probability that a member of the vaccine-eligible population does not have access to an individual who can administer a vaccine using the most complex administration method	**Priority 1**: 1 - [% children delivered by a medical professional who is equivalent to a nurse/midwife or above]; % of children unvaccinated due to limited availability and knowledge of healthcare workers [21, 22] **Priority 2**: % of total population without access to a health facility with any doctors, nurses, emergency medical technicians, or clinical officers [17, 18] **Priority 3**: Average share of staff not in the facilities as observed during one unannounced visit, public facilities [19] **Priority 4**: 1 - [Births attended by skilled health staff (% of total)] [23]
**4. Acceptability of Presentation**	Probability that a member of the vaccine-eligible population (or caregiver) exhibits vaccine non-compliance due to specific characteristics of the vaccine presentation	**Priority 1**: Muslim + Jewish Populations as a % of Total Population [16, 24, 25]. *Potential acceptability issues due to the use of a pork product in the manufacturing process or final vaccine*.
**5. Doses per Container**	Probability that a member of the vaccine-eligible population is refused vaccination due to provider unwillingness to open container	**Priority 1**: % of household survey respondents stating that she or he had taken a child to a health facility for vaccination and the child was not vaccinated because there were not enough children present to open vaccine vial [26].

As described in the Mathematical approach section, a sixth technology-addressable barrier for “Dose Requirements” is included in the calculation structure. We assume that for a vaccine with more than one dose required, an individual must overcome the other five barriers multiple times.

For Vaccine Technology Scores, we created a five-level rubric and used it to map characteristics of vaccine presentations to Vaccine Technology Scores. Table 2 provides the definitions for each category and Table 3 lists the numeric values corresponding with each definition. These numeric values represent the authors’ best guess at the relative importance of each of the five levels in terms of altering the probability that a given barrier will block an individual from vaccination. For the barrier Acceptability of Presentation, we focused on potential acceptability issues due to the use of a pork product.

**Table 2:** Definitions for Vaccine Technology Scores.

Technology-Addressable Barrier	Low	Medium-Low	Medium	Medium-High	High
**1. Vaccine Schedule**	3 or more doses misaligned with existing vaccine schedule	2 doses misaligned with existing vaccine schedule	1 dose misaligned with existing vaccine schedule	Aligned with existing vaccine schedule	No specific schedule must be followed
**2. Temperature Storage Requirements**	Requires unbroken frozen chain (-15C or lower)	Requires unbroken cold chain (2C to 8C)	Requires controlled temperature (CTC; ability to tolerate 40C for at least 3 days)	Minimal temperature storage requirements (e.g., 25C indefinitely)	No temperature storage requirements (hot or cold)
**3. Administration Requirements**	Must be administered by a physician	Must be administered by a formally trained person other than a physician (e.g., nurse)	Administration must be supervised by a formally trained person	Can be administered by a minimally trained health professional (e.g., community health worker)	Can be administered by the patient or caregiver at home
**4. Acceptability of Presentation (Generic)**	Major acceptability issue for a large portion of the population	Minor to medium acceptability issue for a large portion of the population	Medium to major acceptability issue for a small portion of the population	Minor acceptability issue for a small portion of the population	No acceptability issue for the population
**4. Acceptability of Presentation (Presence of Pork Product)**	Pork product used in manufacturing process or in final vaccine; “Haram” (forbidden) or similar religious ruling	Pork product used in manufacturing process or in final vaccine; no religious ruling	Pork product used in manufacturing process; positive religious ruling for most populations	No pork product used in manufacturing process or in final vaccine; vaccine not certified halal for most populations	No pork product used in manufacturing process or in final vaccine; vaccine certified halal for most populations
**5. Doses per Container**	20+ doses	10 doses	5 doses	2 doses	1 dose

**Table 3:** Numeric values for Vaccine Technology Scores.

Technology-Addressable Barrier	Low	Medium-Low	Medium	Medium-High	High
**1. Vaccine Schedule**	0%	25%	50%	75%	100%
**2. Temperature Storage Requirements**	0%	25%	50%	80%	100%
**3. Administration Requirements**	0%	40%	70%	90%	100%
**4. Acceptability of Presentation (Generic)**	0%	30%	40%	70%	100%
**4. Acceptability of Presentation** **(Presence of Pork Product)**	0%	75%	90%	95%	100%
**5. Doses per Container**	0%	20%	40%	75%	100%

For the Calibration vaccine, we used a measles-containing vaccine from Serum Institute of India. This presentation comes in a 10-dose vial, requires unbroken cold storage in 2–8 degrees Celsius, and is administered subcutaneously. Characteristics of this presentation were obtained from the WHO Prequalified Vaccines Database [27]. For the Test vaccine, we used MR-MAP. Because MR-MAP is still in development, we included two different presentations: “Minimum Acceptable” and “Optimal”. We defined these two presentations using the MR-MAP Target Product Profile from the WHO and UNICEF [28].

For current measles vaccine coverage rates, we used national-level data from WHO and UNICEF [29]. We assume that these coverage rates reflect use of the subcutaneous measles vaccine for which we generated Vaccine Technology Scores. We also selected a value for *M*, the highest coverage rate achievable only through changes in vaccine technology. The difference between *M* and 100% coverage represents the population that will remain unvaccinated due to one or more barriers that cannot be addressed by vaccine technologies. For this example analysis, we defined *M* for each country as the maximum coverage among all vaccines (e.g., BCG, DTP) reported in the WHO-UNICEF coverage data set. Finally, we defined the size of the target population for vaccination using population and birth rate data [16, 30].

After compiling the data points described above, we executed the model for the set of Gavi73 countries using the subcutaneous measles vaccine as the “calibration” vaccine and MR-MAP (“Minimum Acceptable” and “Optimal”) as the “test” vaccine.

### Outputs

The first output of the model is a table summarizing the expected coverage rate when using the “test” vaccine and the marginal change in coverage relative to the “calibration” vaccine (Table 4). In our example, these values reflect the aggregate results across all Gavi73 countries. Results suggest that a complete switch from the current subcutaneous presentation to MR-MAP in Gavi73 countries would increase overall measles-containing vaccine coverage by 3.0–4.9 percentage points depending on the final characteristics of the MAP, equating to an additional 2.6–4.2 million additional children vaccinated per year.

**Table 4:** Estimated coverage rates when using MR-MAP in the Gavi73 countries.

Test Vaccine	Estimated Coverage Rate with Test Vaccine	Percentage Point Change Relative to Calibration Vaccine	Total Change in Number of Individuals Vaccinated
MR-MAP “Minimum Acceptable”	83.8%	+3.0	2,600,811
MR-MAP “Optimal”	85.6%	+4.9	4,200,827

**Fig 3:**
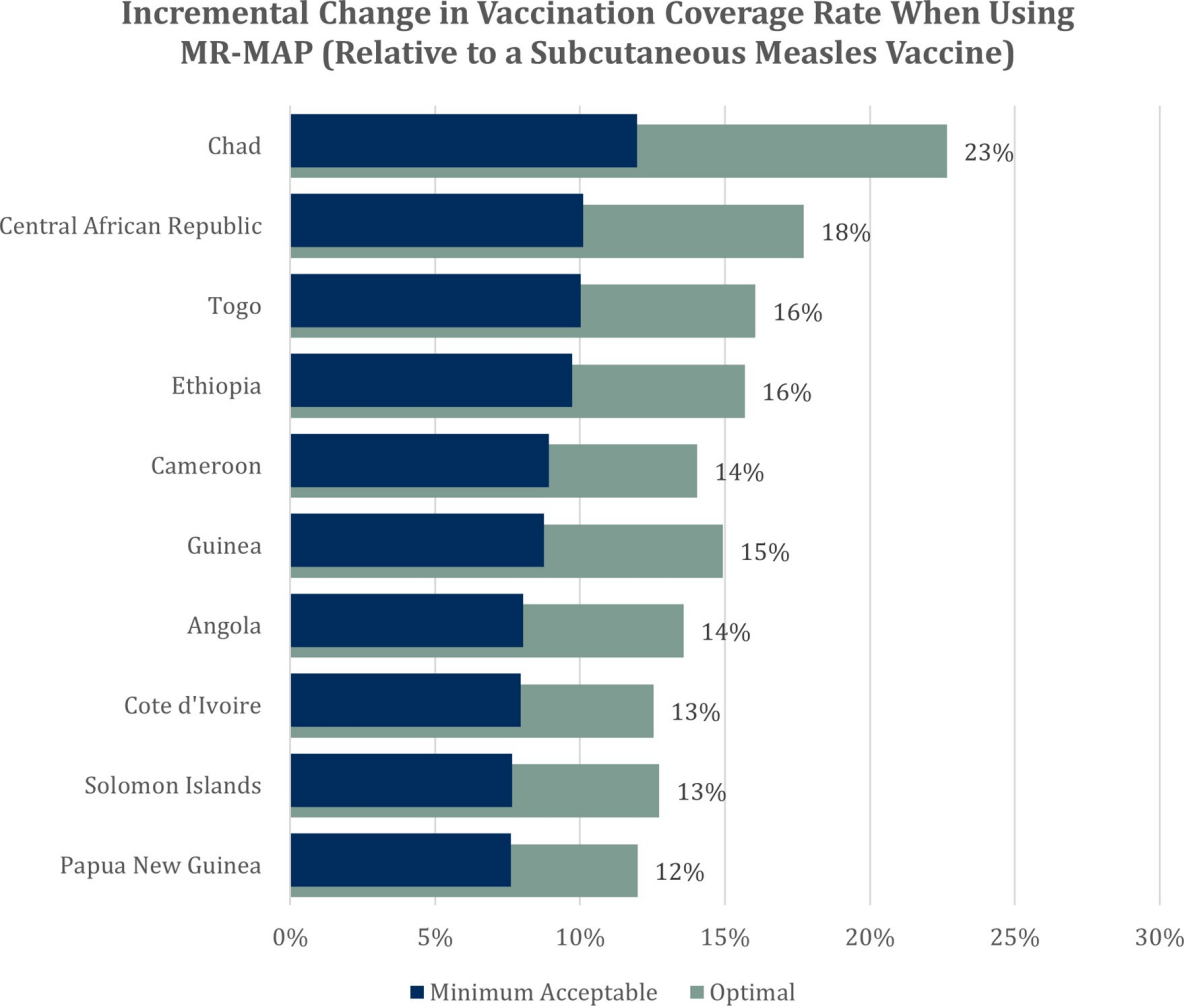
Ten countries with the highest estimated increase in coverage rate when using MR-MAP.

The second output of the model is a chart listing the ten countries or subnational regions in which the expected change in coverage relative to the “calibration” vaccine is the greatest (Fig 3). In our example, while the aggregate incremental change is a maximum of 4.9 percentage points, coverage increases in individual countries could be much greater. Gains in Chad, Central African Republic, Togo, and Ethiopia are estimated to be more than 15 percentage points when deploying a MR-MAP with “Optimal” characteristics.

These results suggest that while MR-MAP could lead to the vaccination of several million additional children per year, this technology alone is insufficient to reach the WHO’s coverage rate target of 90% or more for the first dose of a measles-containing vaccine [31]. Achieving these targets may require long-term interventions that reduce the magnitude of barriers to vaccination or vaccine technologies that further target the specific barriers faced by each population. These results could be combined with data on costs and benefits, such as safety and efficacy, to compare MR-MAP with alternative vaccine presentations.

## Discussion

### Contributions

The method described in this paper provides a practical approach for estimating the coverage rate impact of new vaccine technologies. This method has several benefits. First, it is transparent. Because the calculations are relatively straightforward, the method does not result in a “black box” model. Eq (1), and its expanded form in Eq (12), provides a clear link between each input or assumption and the expected effect on coverage rate. Decision makers can use these equations to identify and address potential areas of disagreement. Second, the method is flexible. The same approach could be used for different administrative levels (e.g., national, regional), technology-addressable barriers, and technology types. Third, the method is systematic. The same approach is used across countries and vaccine technologies, enabling objective comparisons. In our example, we included only MAP. However, this approach would allow a comparison of MAP with other vaccine technologies such as dual-chamber syringes and aerosolized vaccines. Fourth, the method is rapid and low cost. Using publicly available data eliminates the need for lengthy and expensive primary data collection. Rapid generation of outputs could facilitate timely decision-making and enable regular refinement of results as better inputs become available.

We believe this method has long-term utility with vaccine manufacturers, global health organizations, LMIC governments, and other parties involved in creating or purchasing new vaccine technologies. It provides low cost, rapid estimates for informing certain types of decisions about technologies already in development. Moreover, this method could be used for product exploration. One could imagine a slightly different implementation intended to test which characteristics of a new technology are worth developing given a target deployment region and an objective to increase coverage rates.

### Limitations

Our method relies on several assumptions. Some are used due to lack of data and others to maintain simplicity and transparency. Key assumptions include:

○ *The prevalence of technology-addressable barriers to vaccination in the vaccine-eligible population is equal to the prevalence of technology-addressable barriers in the general population*. This assumption is required because few of the data sets describing the prevalence of barriers to vaccination are limited to the vaccine-eligible population.
○ *The increase in the probability that an individual will overcome a technology-addressable barrier is proportional to the Vaccine Technology Score*. This assumption refers to the simple mathematical relationship between the Population Score and Vaccine Technology Score for a given barrier as described in Eq (6).
○ *Each technology-addressable barrier is independent from the other technology-addressable barriers*. This assumption is likely an important over-simplification. Additional data are needed to establish the degree of dependence between barriers in a given country or region. The calibration exercise described in the Mathematical approach section is an attempt to compensate, at least partially, for this over-simplification.
○ *The list of technology-addressable barriers used for analysis is comprehensive*. We assume that this list includes all the technology-addressable barriers in the broader set of all barriers to vaccination.
○ *The observed coverage rate of each Calibration vaccine is attributable to a specific vaccine presentation and its Vaccine Technology Scores*. For a given antigen, there are likely vaccines from several manufacturers available at the same time within a given country or region.
○ *There is a threshold*, *Maximum Coverage*, *beyond which improved vaccine technology will have no effect on coverage rate*. We assume that there is some proportion of vaccine-eligible individuals that faces barriers which cannot be addressed by new technologies (e.g., vaccine hesitancy due to distrust of government).

Another limitation is the lack of data for full validation. Even in the absence of full validation, we hope that this method will be useful, especially for decisions that only require rank ordering vaccine technologies based on coverage rate estimates.

A third limitation is the inability of the method to handle extreme values of inputs. If there is a large gap between the value of Maximum Coverage (*M*) and the current coverage of the “calibration” vaccine (*C*_*c*_), there will be a large scaling factor and a coverage rate for the “test” vaccine (*C*_*t*_) greater than 100%. In this case, our method overestimates the importance of vaccine technology. One potential explanation is that the Calibration vaccine is poorly aligned with the vaccine used to define Maximum Coverage concerning the level of investment and the role of that investment in addressing barriers unrelated to vaccine technologies. Another potential explanation is that the Calibration vaccine has not been fully deployed and integrated. Our method does not distinguish technology impact from the effects of rollout and integration. If the analysis from a specific country and sub-national region falls into this special case, its outputs are excluded from the multi-country results. Of the 73 countries included in the example analysis, only Syria fell into this category. Its birth cohort represents approximately 0.5% of the total cross-country birth cohort.

### Comparison to alternatives

To our knowledge, there are few alternatives for generating coverage rate estimates for new vaccine technologies using publicly available data. For the potential alternatives identified, such as PATH’s Vaccine Technology Impact Assessment tool, information available in the literature and other public sources is insufficient for comparison.

### Potential improvements

There are several changes to the proposed method and its implementation that could improve the accuracy of coverage rate estimates. We believe the most important changes are those related to the assumptions mentioned previously:

○ Using more accurate input data, such as Population Score proxies that are better aligned with the ideal definitions or are specific to the target population for vaccination.
○ Refining the Vaccine Technology Score rubric based on known relationships between vaccine characteristics and barriers to vaccination. In the absence of better data to refine the rubric, adjustment and validation by experts could also increase the accuracy of these scores and thus the coverage rate estimates. We began this process of expert review in the context of a WHO-convened working group but were ultimately unsuccessful due to limited availability of experts and shifting of priorities to Covid-19 response.
○ Defining a more complex mathematical relationship between Population Scores and Vaccine Technology Scores. For example, an alternative relationship could be, “the lower the Population Score, the higher the Vaccine Technology Score needs to be to affect the final probability of overcoming a specific barrier." This type of relationship could better reflect the challenge of overcoming a barrier for the portion of the population in which it persists.
○ Restructuring the calculations to account for conditional probability and obtaining data on the degree of overlap between technology-addressable barriers.

## Conclusions

The Covid-19 pandemic has highlighted the presence of supply- and demand-side barriers to vaccination in both high-income countries and LMICs. As vaccines for Covid-19 are developed and deployed, we see examples of how vaccine characteristics can affect coverage rates. The need for ultra-cold chain and injection by trained medical staff places a heavy burden on already straining supply chains and health systems, limiting access and slowing roll out in certain regions. A requirement for two doses instead of one can lead to missed follow-up appointments and partial vaccination. Mechanisms such as using mRNA to generate specific proteins can reduce vaccine acceptability and uptake in some populations, despite demonstrated efficacy.

Although widespread vaccination is a pillar of epidemic control for Covid-19 and other diseases, there are few resources available to quantify the relationship between vaccine presentation, target population characteristics, and coverage rate. In this article, we present and implement a method to address this gap. Our method is transparent, flexible, systematic, rapid, and low cost. It emphasizes the interaction between vaccine characteristics and deployment context. While an innovative vaccine that fails to address the most prevalent barriers could have little impact on coverage, a vaccine specifically targeted to those barriers could have an outsized effect.

Although our method has several limitations, we believe it provides estimates sufficient for many use cases, especially those requiring only rank ordering based on coverage rates or the relative magnitudes of those rates. This type of information is necessary for the cost-benefit or cost-effectiveness analysis that allows vaccine manufacturers and purchasers to make objective comparisons between options and obtain the most value for money. Used in conjunction with other health economic approaches, this method could support a diverse set of activities such as refining a research pipeline, determining the composition of a vaccine portfolio that maximizes coverage rates within a given budget, and targeting certain vaccine presentations to the countries in which they will have the greatest impact.

Finally, we strongly believe that methods and tools developed through public funding or used to influence the allocation of public funds should be made publicly available in a timely manner. The ability of the public to scrutinize these methods and tools is especially important when the organizations using them can significantly influence a particular market. This article represents what we hope is one of many efforts to move the global health space towards increased transparency.

## Supporting information

S1 File Excel model used for example application. (XLSM)
